# Exosome-Mediated Genetic Information Transfer, a Missing Piece of Osteoblast–Osteoclast Communication Puzzle

**DOI:** 10.3389/fendo.2017.00336

**Published:** 2017-11-27

**Authors:** Pengbin Yin, Houchen Lv, Yi Li, Yuan Deng, Licheng Zhang, Peifu Tang

**Affiliations:** ^1^Department of Orthopedics, Chinese PLA General Hospital, Beijing, China

**Keywords:** exosome, intercellular communication, microRNA, osteoblast, osteoclast

## Abstract

The skeletal system functions and maintains itself based on communication between cells of diverse origins, especially between osteoblasts (OBs) and osteoclasts (OCs), accounting for bone formation and resorption, respectively. Previously, protein-level information exchange has been the research focus, and this has been discussed in detail. The regulative effects of microRNAs (miRNAs) on OB and OC ignite the question as to whether genetic information could be transferred between bone cells. Exosomes, extracellular membrane vesicles 30–100 nm in diameter, have recently been demonstrated to transfer functional proteins, mRNAs, and miRNAs, and serve as mediators of intercellular communication. By reviewing the distinguishing features of exosomes, a hypothesis was formulated and evaluated in this article that exosome-mediated genetic information transfer may represent a novel strategy for OB–OC communication. The exosomes may coordinately regulate these two cells under certain physiological conditions by transferring genetic information. Further research in exosome-shuttered miRNAs in OB–OC communication may add a missing piece to the bone cells communication “puzzle.”

## Introduction

It is well documented that the skeletal system functions and maintains itself based on communication between cells of diverse origins. Osteoblasts (OBs) and osteoclasts (OCs), accounting for bone formation and resorption respectively, are of importance for bone homeostasis. The coordinated functions of these two cell lines especially during bone remodeling contribute to a dynamically stable bone mass and quality. Previously, protein-level information exchange has been the research focus in OB–OC communication and has been well documented. So far three modes of communication have been discovered, which are direct contact (i.e., membrane-bound ligands and gap junctions), diffusible paracrine factors (i.e., growth factors and cytokines), and OC absorbing growth factors that are deposited in bone matrix by OBs (1). However, genetic-level information exchange between OB and OC remains a mystery.

Genetic molecules such as microRNAs (miRNAs) that display a function of coordinately regulating OB and OC have been observed (2, 3). miRNAs, small strands (~22 nucleotides) of RNA, have been demonstrated to play crucial roles in bone cell differentiation and function, thereby affecting bone development. They may also play a causal role in various bone diseases (4). A large number of miRNAs are found to exist in both OBs and OCs, and they coordinate these two types of cells to maintain bone homeostasis under different conditions (5). For instance, miR-31, which inhibits OB differentiation, has been found to have a positive effect on OC differentiation, during the initiation phase of bone remodeling (6, 7). In addition, miR-146a plays a positive role in OBs but a negative role in OCs, which is of importance for the transition phase of bone remodeling (8, 9). Unfortunately, miRNA has been recognized to be unstable in the extracellular space. Therefore, studies on the role of miRNAs in the skeletal system focus on intracellular function by observing OBs and OCs separately. No prior evidence has shown that miRNA could transfer between OB and OC, thereby participating in bone cell communication.

## Hypothesis

Recently, exosomes, extracellular membrane vesicles 30–100 nm in diameter, have been demonstrated to transfer functional proteins, mRNAs, and miRNAs to neighboring cells and serve as mediators of intercellular communication (10). This novel finding sheds light on miRNA’s transportation between different cells. Noticeably, a review by Vlassov et al. emphasizes that exosomes provide necessary protective packaging for miRNAs, which protects the miRNAs from degradation (11). Moreover, some very recent studies, albeit limited in the number, may show the same phenomenon of miRNAs transportation among bone cells (12–14). Given this, our hypothesis is that exosomes may represent a novel strategy for OB–OC communication, by which they coordinate functions of these two cells under certain physiological conditions by transferring genetic information.

## Evaluation of Hypothesis

Secreted vesicles known as exosomes were first discovered nearly 30 years ago (15). The term exosome was used to describe small membrane vesicles formed by vesiculation of intracellular endosomes and released by exocytosis (16). Initially, studies into the role of exosomes were limited as they were considered little more than cellular garbage cans acting to discard unwanted molecular components (17). However, renewed interest in exosomes followed recent reports showing that the vesicles act like signaling packages containing cell-specific collections of proteins, lipids, and genetic material, which are transported to other cells where they alter function and physiology (18).

### Exosomes Participate in Intercellular Communication

The first evidence showing exosomes as an active player in intercellular communication came from Raposo et al. who identified that exosomes present the MHC–peptide complexes to specific T cells, participating in adaptive immune responses (19). Further research by the same group demonstrated that dendritic cells also secrete exosomes bearing functional MHC–peptide complexes, which could promote induction of antitumor immune responses in mice *in vivo* (20). Successive research has further recognized exosomes and their messenger role as a novel mechanism of intercellular communication in various physiological and pathological processes, including vascular remodeling, tumor metastasis, airway remodeling, and neuron signal transduction, etc. (21–24). In addition, given these findings, a close association (either positive or negative) between exosomes and various diseases has also been observed, such as cancer, osteoarthritis, atherosclerosis, primary hypertension, Alzheimer’s disease, and Parkinson’s disease (25–30).

### Exosome Selectively Package Genetic Information

In 2007, Lötvall et al. first described the presence of mRNA and miRNA inside exosomes (31). Ignited by this discovery there was an exponential increase in the numbers of papers on exosomes. By comparing the mRNAs in exosomes to those in their parental cells, the identified mRNA in exosomes was approximately 8% of the mRNA detected in the parent cells. The gene profile analysis of these mRNAs displayed essential differences in the level of mRNA transcripts from exosomes versus their parental cells. The most abundant transcripts in the exosomes were generally different from the most abundant transcripts in the parental cells. Apparently, a subset of some specific targeting mRNA sequences was controllably enriched in the released vesicles (31). Such observations rule out the original idea that mRNA in exosomes results from random contamination. When it comes to miRNAs, research also showed that the miRNA repertoire of exosomes differs from that of the producer cell and what is more, miRNAs patterns in exosomes are associated with cellular processes such as exocytosis, tumor formation and angiogenesis (32–35). Similar phenomena are further supported by several reports for example in exosomes from ovarian tumor cells, T cells, and dendritic cells (36–38). Moreover, the fact that exosomes lack almost all ribosomal RNA, which is the most enriched RNA species in cells, also supports “selective package” rather than “random contamination.” Given these findings, a sorting mechanism must exist that provides exosomes with a unique subset of RNA. Further investigation discovered various pathways controlling the specific sorting of RNAs into exosomes. For instance, research by Villarroya-Beltri et al. indicated that Sumoylated heterogeneous ribonucleoprotein A2B1 controls the sorting of miRNAs into exosomes through binding to specific motifs, namely, EXOmotif (GGAG) (39). In addition, Annexin-2 is another protein that might play a role in RNA sorting into exosomes (40). As for mRNAs, evidence has shown that exosomal mRNAs seems to be enriched in 3′UTR fragments, which might be important for the sorting of specific mRNAs into these vesicles (41). However, as pointed out in a recent review, the exact mechanism has yet to be fully understood (42).

### Exosome-Shuttled RNAs’ Function in Target Cells

The Jan O. Lötvall group not only confirmed the existence of mRNAs and miRNAs in exosomes but also demonstrated, at least *in vitro*, that some mRNA present in exosomes could be translated into proteins in target cells, which implied that exosomes can transfer functional genetic information (31). Thereafter, the functionality of transferred exosome-shuttled mRNAs and miRNAs is shown by other studies using a luciferase reporter gene assay. Cells were transfected using a vector that codes for a luciferase reporter gene in conjunction with a sequence complementary to the target miRNA found in their exosomes and a decrease of luciferase activity in the cell was given as an indication of a successful transfer and function (37). More importantly, as increasing attention has been given to this area of study, the functionality of the exosome-shuttled RNA has further been confirmed by the fact that the RNAs (both mRNAs and miRNAs) actually exert biological effects in recipient cells. For instance, stem cell-derived exosomes have been shown to contain specific miRNAs, such as miR-21, miRNA-494, and miRNA-183-5p, which are of great importance for tissue regeneration (43). These reports, combined with the findings described earlier, indicate that exosomes play a crucial role in intercellular communication by selectively transferring functional genetic information between cells. This communication between cells through exosome-shuttled RNA is one of the key processes in both maintenances of normal physiological processes and disease mechanisms.

## Potential Role of Exosome in OB–OC Communication

### Exosomes, Missing Piece of OB–OC Communication Puzzle

As mentioned earlier, three modes of OB–OC communication have been recognized, which mainly focus on protein exchange. However, as accumulating evidence shows that miRNAs coordinately regulate OB and OC, especially during bone remodeling in which intercellular communication play a key role (44), the question is ignited as to whether these miRNAs can somehow be transferred between bone cells, influencing the balance of bone formation and resorption. Enlightened by the recent research regarding exosomes, it may not be unreasonable to make a hypothesis that exosomes may provide a payload to miRNAs so as to protect them from degradation by ubiquitous RNases, extreme temperatures, and pH levels in extracellular environment and pave the way for intercellular communication between OB and OC.

Recently, research has revealed the involvement of a number of bone-derived miRNAs-bearing exosomes in the regulation of bone remodeling. miR-503-3p was shown to be upregulated in OB-derived exosomes during mineralization. It was previously found to be expressed in OCs, and it inhibits RANKL-induced OC differentiation by regulating RANK expression (45). In addition, miR-148a, which is known to promote osteoclastogenesis by targeting the MAFB gene, was found to be upregulated in HBMSC exosomes (46, 47). Based on these observations, reasonable deduction could be made that miR-503-3p and miR-148a may probably exert physiological effects when exosomes containing these two miRNAs are incubated with recipient cells. In addition, a review by our group also provides a summary of bone-derived exosomal miRNAs that may represent a means of communication between OBs and OCs. Noticeably, recent research by Sun et al. and Li et al. both suggested that OC-derived miR-214 can be transferred through exosomes, thereby inhibiting OB activity. In both these studies exosomes derived from OCs were incubated with OBs (12, 13). These two studies provide the first direct evidence to support the hypothesis that functional genetic information could be transferred through exosome between OB and OC, albeit further investigations are needed to confirm the results in humans.

Of note, matrix vesicles, discovered in 1970, have been recognized as unique extracellular membrane-bound microparticles that provide initial sites for mineral formation in endochondral bone (48). Observations into the cargo of matrix vesicles showed that they contain various intracellular components derived from their parental cell, including enzymes, membrane proteins, and lipids. It is plausible to expect that these molecules would play crucial roles in not only the mineralization of the matrix but also cell–cell communication. Nevertheless, little attention was given to the intercellular role of matrix vesicles until the findings in exosomes, or more generally in all secreted vesicles were published leading to the blossoming of the topic into a full-fledged field of research—that of vesicle-modulated cell–cell communication. In fact, a recent review by Shapiro et al. argues that, based on the analyses of size, morphology and lipid and protein content, matrix vesicles are generated in cells as microparticles (endosomes), which are subsequently released from the cell as exosomes (49). Although this argument may require further evidence at least it suggests that a totally different intercellular communication involving extracellular vesicles (EV) may deserve further attention as a missing piece of OB–OC communication.

### Major Questions Remain in Verifying the Hypothesis

First, important questions still remain regarding intercellular communication involving the transfer of genetic information. Earlier studies suggested that genetic material, mainly in the form of regulatory RNAs, can be exchanged between mammals’ cells. Transfer of genetic material adds an exciting and novel dimension to the cell–cell communication models in complex organisms. However, the questions such as whether transfer occurs at endogenous levels of these molecules, and the physiological importance of this movement, especially in specific tissue or biological process still remain (50).

In addition, a large number of studies have shown the effects of exosomes on target cells, but as suggested by Théry et al. in a recent review in cell (51), a major challenge for understanding how exosomes may support both physiological and pathophysiological processes is being able to demonstrate *in vivo* EV transfer between cells. *In vitro* approaches based on overexpression of mRNA or miRNA in target cells combined with the PCR evidence of expression of certain mRNA or miRNA in exosomes released by the parental cells suggest that these molecules are functionally active in certain processes, but a direct demonstration that functional exosome-mediated RNAs transfer is the relevant mechanism in certain biological processes is still difficult to achieve. An inhibition of EV biogenesis *in vivo* would be ideal; however, these experiments are quite difficult to accomplish.

In regard to OB–OC communication, the evidence for exchange of genetic material between these two cells remained undiscovered until recent articles showed miRNA-bearing exosomes may be involved. As included in our hypothesis, research by Sun et al. and Li et al. both suggested that OC-derived miR-214 can be transferred through exosomes, thereby inhibiting OB activity, by directly incubating exosomes derived from OC with OB. These studies shed light on the possibility of transfer of genetic material across the bone cells, therefore lay the groundwork for the hypothesis that exosomes may play an important role in the process. Nevertheless, major questions on whether exosomes represent a missing piece of OB–OC communication puzzle still remain. Further information is needed on: (1) whether transfer of genetic material occurs *in vivo* at endogenous levels of miR-214 or other RNAs and (2) the physiological relevance of this exosome-intermediated genetic transfer movement, as compared with protein-level communications. Finally, the loss-of-function and the gain-of-function experiments were performed in the published studies by using a knockout or a knock-in of miR-214 model. These experiments proved that miR-214 did exert a crucial effect in bone cells. However, no direct demonstration of functional exosome-mediated transfer, such as results from applying an inhibitor/promotor of exosome biogenesis *in vivo*, is available.

### Importance to Confirm the Hypothesis

Confirmation of the exosome-intermediated miRNAs transportation as an intercellular communication strategy may add a missing piece to the bone cells communication “puzzle” of how genetic-level information is exchanged among diverse cell origins. In addition, it is noteworthy that the burden of bone diseases like osteoporosis keep increasing as the general population ages (52). Drugs targeting a single type of cell may end up affecting others, due to the disruption of intercellular communication, thereby causing side effects. Interestingly, the effect that a single type of miRNA may exert on OBs and OCs, respectively, can be variable, including positive on both cells, positive on OBs while negative on OCs, negative on OBs while positive on OCs, and negative on both cells (53). Research into finding certain miRNAs that exert desirable effects on both OBs and OCs and fully illuminating the exosome-intermediated miRNAs communication between bone cells may provide novel therapeutic targets or at least be a good place to start.

## Author Contributions

PT and LZ made major contributions to the conception of the work. PY, HL, and YL drafted the manuscript. YD revised the manuscript.

## Conflict of Interest Statement

The authors declare that the research was conducted in the absence of any commercial or financial relationships that could be construed as a potential conflict of interest.
